# Visits to Private Asylums

**Published:** 1904-09-10

**Authors:** 


### VISITS TO PRIVATE ASYLUMS.

By Our Rambling Reporter.

#### HALLIFORD HOUSE, SUNBURY-ON-THAMES.

Halliford House is about sixteen miles from London, and four from Hampton Court. It is easily reached by the South Western Railway from London, or from the West of England by the Reading and Richmond line. It is just over a mile from Sunbury Station and three miles from Walton Station.

The house stands about a hundred yards from the road. The entrance gates are kept unlocked, and immediately on entering we see the lawns, and shrubberies, and gardens, and the fine old timber, the latter being especially remarkable. The grounds extend to forty-five acres, and as the Commissioners in Lunacy remarked in one of their reports, "afford great facilities, as well as inducements, for walking exercise."

The Asylum is licensed for thirty patients, fifteen of each sex, and it has been an asylum for sixty-three years. The present medical superintendent has been the resident proprietor for about thirteen years; and previous to taking up that position he was medical officer in a lunatic hospital where every variety of insanity was under treatment. Like most old mansions, Halliford House has had to go through the process of being altered and added to. The ladies now live in the older or original part, and the men have a detached block to themselves.

The main entrance to the building we also found unlocked, and although nearly thirty patients are in residence, it must be said that the private house appearance of the asylum has been fairly well retained. The sitting-rooms are not large, but they are cosy and have pleasant outlooks, several having French windows opening on to the lawns and the ladies occupying these rooms can go out and in as they please. There are no dormorities in the ordinary asylum meaning of the word. Every patient has a room to herself or himself, but some of the rooms have two beds in case the presence of a nurse or attendant should be thought desirable. All the rooms are nicely furnished, and have the bright, cheerful look so common in asylums nowadays. Careful precautions have been taken to render the escape of patients easy in case of fire occurring in the house. To get private patients to employ themselves in any kind of outdoor work is always difficult, as they have never been accustomed to outdoor work; hence games have to take the place of employment, and at Halliford House, tennis, croquet, and billiards are indulged in. The kitchen gardens produce all the vegetables and fruit required for the patients, and, what is of more importance, a sufficient number of cows is kept to produce the milk and butter.

The recovery rate is high. The average for the last two years has been 66 per cent. of the admissions, while the death-rate has been only 6 per cent. of the average number resident. The staff seems quite sufficient for the number and class of patients; in fact, it is as numerous as the patients.

The payments for patients range from two and a half to five guineas a week. There are many private asylums which we should not like to see enlarged, chiefly those having five or six patients only; but when an asylum already contains thirty patients, when plenty of land belongs to the house, and when no chance exists of its ever being overlooked by troublesome neighbours, we are satisfied that permission to enlarge should be granted. Such a case is that of Halliford House. It should certainly be allowed to double its numbers; not for the interest of the proprietor, but for the interests of a large class of the insane.

